# Deregulation of apoptosis mediators' p53 and bcl2 in lung tissue of COPD patients

**DOI:** 10.1186/1465-9921-11-46

**Published:** 2010-04-27

**Authors:** Marianna Siganaki, Anastasios V Koutsopoulos, Eirini Neofytou, Eleni Vlachaki, Maria Psarrou, Nikolaos Soulitzis, Nikolaos Pentilas, Sophia Schiza, Nikolaos M Siafakas, Eleni G Tzortzaki

**Affiliations:** 1 Laboratory of Molecular and Cellular Pulmonology, Medical School University of Crete, Greece; 2 Department of Pathology, Medical School, Democritus University of Thrace, Alexandroupolis, Greece; 3 Department of Thoracic Medicine, University Hospital of Heraklion Crete, Greece; 4 Laboratory of Clinical Virology, Medical School University of Crete, Greece; 5 Department of Anesthesiology, "G. Gennimatas" Hospital Athens, Greece

## Abstract

Abnormal apoptotic events in chronic obstructive pulmonary disease (COPD) subvert cellular homeostasis and may play a primary role in its pathogenesis. However, studies in human subjects are limited.

p53 and bcl2 protein expression was measured by western blot on lung tissue specimens from 43 subjects (23 COPD smokers and 20 non-COPD smokers), using beta-actin as internal control. Additionally, p53 and bcl2 expression patterns were evaluated by immunohistochemistry in formalin-fixed, paraffin-embedded lung tissue sections from the same individuals.

Western blot analysis showed statistically significant increased p53 protein levels in COPD smokers in comparison with non-COPD smokers (p = 0.038), while bcl2 protein levels were not statistically different between the two groups. Lung immunohistochemistry showed increased ratio of positive p53-stained type II pneumocytes/total type II pneumocytes in COPD smokers compared to non-COPD smokers (p = 0.01), whereas the p53 staining ratio in alveolar macrophages and in lymphocyte-like cells did not differ statistically between the two groups. On the other hand, bcl2 expression did not differ between the two groups in all three cell types.

The increased expression of pro-apoptotic p53 in type II pneumocytes of COPD patients not counterbalanced by the anti-apoptotic bcl2 could reflect increased apoptosis in the alveolar epithelium of COPD patients. Our results confirm previous experiments and support the hypothesis of a disturbance in the balance between the pro- and anti-apoptotic mediators in COPD.

## Introduction

COPD is a leading cause of morbidity and mortality among the adult population [1]. It is a cigarette smoking-related disorder characterized by chronic inflammation of the airways and progressive destruction of lung parenchyma leading to airway remodeling and pulmonary emphysema [1]. Several mechanisms contribute to the pathogenesis of COPD, including influx of inflammatory cells into the lung, disruption of the balance between proteolytic and anti-proteolytic activity and oxidative stress [1]. Recent data described abnormal apoptotic events as the fourth important mechanism involved in the destruction of pulmonary tissue in COPD [2-7]. There are two main apoptotic pathways the extrinsic (receptor-mediated) and the intrinsic (mitochondria-mediated) pathway [2-7].

The intrinsic pathway of apoptosis may be triggered by both internal and external stimuli and includes many mediators, which either promote or inhibit the process [6,7]. The most representative regulators of the mitochondria-mediated pathway are p53, an inducer of apoptosis, and bcl2, a molecule with the opposite function [8-10].

P53 is a tumor suppressor protein that maintains genomic integrity during cellular stress and protects from DNA damage either by stimulating DNA repair or by initiating apoptosis when DNA damage is beyond a certain threshold [8,9,11].

Bcl2 family of proteins is situated upstream of the apoptotic pathway defending from irreversible cellular damage providing a pivotal decisional checkpoint for cells after a death stimulus [10,11]. Both pro- and anti-apoptotic bcl2-family members have been identified. Bcl2 is a mitochondrial outer membrane permeabilization protein which functions by extending cellular survival via inhibition of a variety of apoptotic deaths, whether these are p53 dependent or independent [6-11].

Inhaled oxidants from cigarette smoking and increased amount of reactive oxygen species (ROS) generated by various inflammatory cells in the airways of COPD patients, leads to oxidative DNA damage of host cells [12] and subsequently triggers the intrinsic apoptotic cascade mediated by an atypical immune response with the predominance of CD8+ cytotoxic cells [7,12,13]. Furthermore, recent studies suggested that a disruption of the balance between apoptosis and replenishment of lung structural cells might be involved in the pathogenesis of COPD [7,14-16].

To the best of our knowledge, no previous reports have examined the expression pattern of pro-apoptotic p53 and anti-apoptotic bcl2 mediators, both implicated in the intrinsic pathway of apoptosis, in lung specimens of smokers with and without COPD. The results of this study revealed an imbalance between pro- and anti-apoptotic mediators in COPD.

## Materials and methods

### Study Subjects

The study was performed on lung tissue specimens from 43 male subjects who underwent open lung surgery for the excision of solitary pulmonary nodule. Subjects were divided in two groups:

A) 23 COPD smokers, according to GOLD criteria [1].

B) 20 non-COPD smokers

Smokers were defined as subjects who had a history of at least 20 pack-years of cigarette smoking [17]. All subjects underwent routine pulmonary function testing, measurements of arterial blood gases, and chest radiography. The GOLD spirometric classification of COPD severity, based on post-bronchodilator FEV_1 _was used for the diagnosis of COPD [1]. All COPD patients participated in this study were GOLD stage II (FEV_1_/FVC<0.70, with 50% ≤ FEV_1 _≤ 80% predicted), (Table 1). COPD patients were treated with a long (tiotropium) or short-acting (ipratropium) inhaled anticholinergic [1]. In order to achieve the best possible baseline function peri-operative and to decrease risk of postoperative complications, COPD group received twice-daily low dose inhaled corticosteroids for 10 days in total (2-3 days before surgery and continued until hospital discharge). The data on drug regimen of the patients are shown in the table 1.

**Table 1:** Anthropometric characteristics, spirometric values and drug regimen of the subjects.

	COPD smokers	Non-COPD smokers	*P value
**Number**	23	20	

*Sex (M/F)*	23/0	20/0	

*Age (years)*	64 ± 7*	57 ± 10*	0.03

*Smoking (P-Y)*	60 ± 21*	50 ± 27*	NS

*FEV1 (% pred.)*	64 ± 16*	95 ± 13*	0.0001

*FVC (% pred)*	80 ± 18*	91 ± 13*	0.02

*FEV1/FVC (%)*	63 ± 6.5*	82 ± 5*	0.0001

*IPRATOPIUM* *or* *TIOTROPIUM*	20 mcg, ×3/day Once daily	NA	

*ICS*	100 mcg ×2/day	NA	

M/F: Male/Female

P-Y: pack years of smoking (mean ± SD)

FEV1: forced expiratory volume in 1 second (mean ± SD)

FVC: forced vital capacity (mean ± SD)

ICS: inhaled corticosteroids (BUDESONIDE or BECLOMETHAZONE or FLUTICAZONE)

NS: non-significant

NA: not applicable

Informed consent was obtained from all subjects participating in the study, and the study was approved by the Medical Research Ethics Committee of the University Hospital of Heraklion, Crete.

### Tissue preparation

Human lung tissue samples were collected from all subjects from an uninvolved segment of the subpleural parenchyma at least 5 cm away from the solitary nodule. Samples were immediately frozen in liquid nitrogen and stored at -80°C until use. For immunostaining, additional tissue blocks were fixed in 10% formalin for at least 24 hours. After fixation, each tissue block was embedded in paraffin and sections 5 μm thick were cut following routine procedures.

### Western blot

Western blot detection of p53, bcl2 and b-actin, which was used as internal control, was performed using standard protocols. In detail, lung tissue specimens from all subjects were homogenised in order to obtain the corresponding protein extracts. The protein lysate was added to 1/3 volume of SDS-preparation buffer (NuPAGE LDS 4× LDS Sample Buffer, Invitrogen Corp., USA). Sample preparations of each lung protein sample (50 ng) were separated by 12.5% SDS-polyacrylamide gel electrophoresis. The proteins were then transferred electrophoretically from the gels to a nitrocellulose membrane. Membranes were incubated with either mouse anti-p53 monoclonal antibody (X77 Santa Cruz Biotechology Inc, USA) or rabbit anti-bcl2 polyclonal antibody (C21 Santa Cruz Biotechology Inc, USA). After applying a secondary antibody, immunodetection was performed with enhanced chemiluminescence, detected on X-ray films (Fuji films). The mouse anti-actin antibody (MAB 1501, Chemicon, Temecula, CA) was used in order to normalize p53 and bcl2 expression. Films were scanned and the protein lanes were quantified using the Photoshop CS2 image analysis software (Adobe Systems Inc., CA).

### Immunohistochemistry

**Figure 1:**
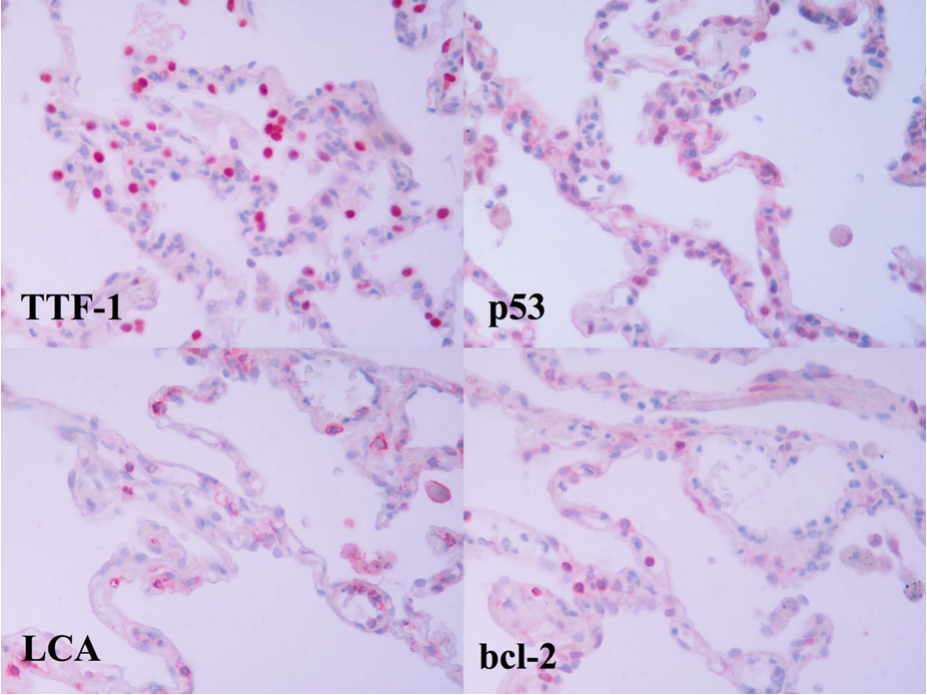
**Positive TTF-1 and p53 immunostaining in type II pneumocytes in serial sections from a COPD patient**. Positive LCA and bcl2 immunostaining in lymphocyte-like cells in serial sections from a COPD patient (400× magnification).

Immunostaining for p53 and bcl2 was carried out using standardized protocols. Tissue samples were fixed in 10% formalin and embedded in paraffin. 5 μm thick serial tissue sections, were obtained and mounted in Superfrost/Plus glass slides (Fischer Scientific). Deparaffinization was performed by heating the sections for 1 h at 60°C followed by washing three times for 5 minutes in xylene, then washing in 100%, 95%, 80%, 70% ethanol three times for 5 minutes, and finally rinsing with distilled water. Incubation of the primary antibody was followed by detection with a labelled streptavidin-biotin peroxidase kit (DAKO LSAB kit). Sections were counterstained blue with haematoxylin. Positive (breast carcinoma with known positivity) and negative (omission of primary antibody) controls were used for each antibody. Given that alveolar macrophages may resemble type II pneumocytes, we used TTF-1 staining against type II pneumocytes, as positive control (Figure 1), using the monoclonal mouse anti-TTF-1 antibody (Santa Cruz Biotechnology, Inc) on adjacent serial sections [18]. Likewise, for the identification of lymphocytes we have used LCA stain (lymphocyte common antigen; DAKO Carpinteria, CA, USA), (Figure 1). Yet, five μm sections were sufficiently thin to guaranty that each cell was present in adjacent sections since the diameter of the type II pneumocytes and alveolar macrophages is much higher than 15-25 μm [18-20].

The evaluation of total PN II (columnar alveolar lining cells), AM (irregularly distributed in the alveoli with foamy cytoplasm and indented nuclei) and LYM (scattered spherical ovoid cells with dense nuclear chromatin and high nuclear/cytoplasmic ratio) in the stained sections was performed using a digital camera (Sony) in a multiread light microscope (Olympus), at 40× magnification by two scientists experienced in lung pathology (AVK and MS). The inter-observer variability of measurements was expressed as the % coefficient of variation. The inter-observer coefficient of variation was less than 10%. Twenty microscopic fields under a semitransparent grid of horizontal lines spaced at 1-mm intervals were used for cell counting. Results were expressed as cells per mm^2^.

### Statistical analysis

Statistical differences between COPD patients and non-COPD subjects, their smoking status, anthropometric and spirometric values, and the expression levels of each apoptotic marker were evaluated with Mann-Whitney and Spearman test using the SPSS 17.0 statistical software package (SPSS Inc; Chicago, IL). A p-value of < 0.05 was considered to be significant.

## Results

### Clinical characteristics of the subjects

The anthropometric characteristics and spirometric values of smokers with or without COPD are shown in Table 1. As expected from the selection criteria, smokers with COPD had a significant lower value of FEV1 (pred %) and FEV1/FVC ratio (%) than non-COPD smokers.

### Western blot

**Figure 2:**
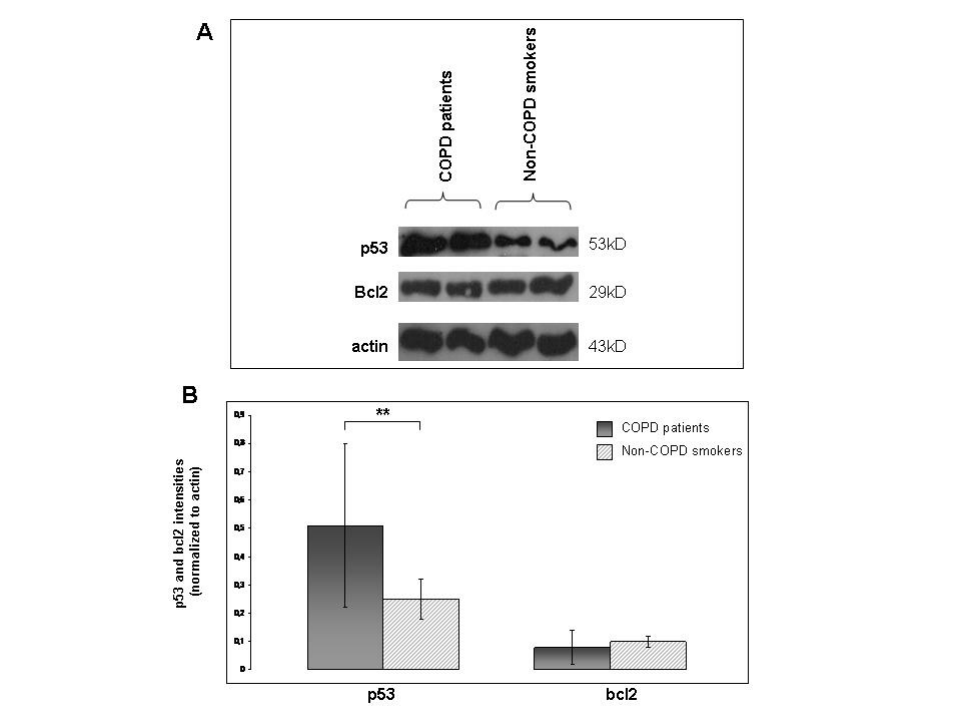
**(A): Representative western blots of p53, bcl2 and b-actin in human lung tissues from two COPD and two non-COPD smokers**. **(B)**: Quantitative analysis (mean ± SD) of p53 and bcl2 protein levels in COPD smokers in comparison to non-COPD smokers. **Statistically significant (p < 0.05).

Western blot analysis revealed statistically significant increased p53 protein levels in COPD patients compared with non-COPD smokers (0.51 ± 0.29 versus 0.25 ± 0.07, p = 0.03), (Figure 2). On the contrary, bcl2 protein levels did not differ statistically between the study groups (0.08 ± 0.06 versus 0.10 ± 0.02, p = 0.52), (Figure 2).

### Immunohistochemistry

#### p53 immunostaining

**Figure 3:**
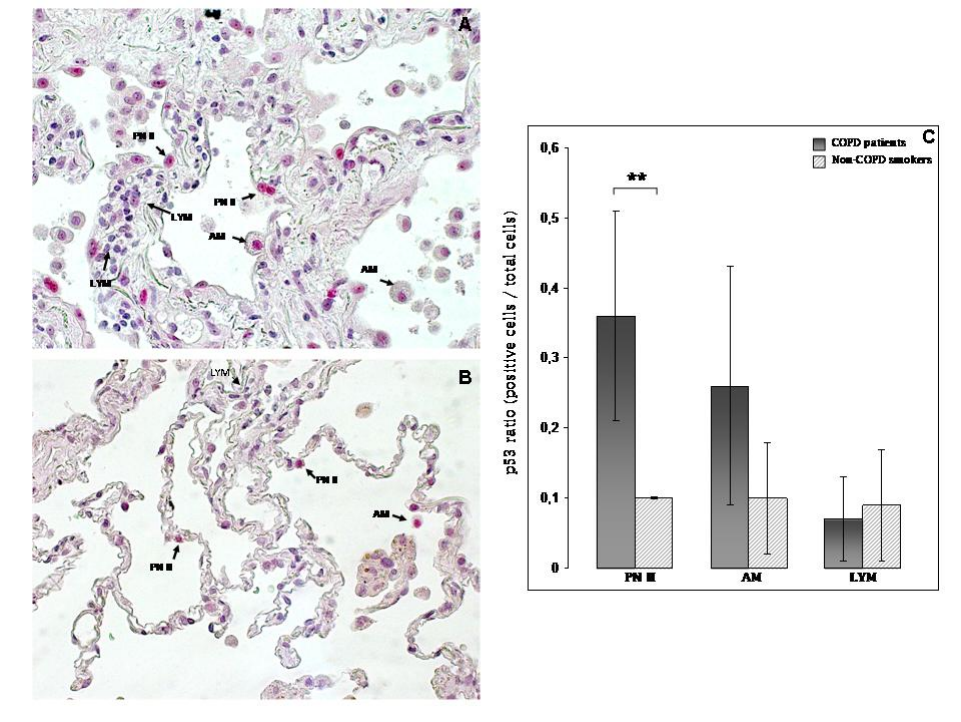
**Immunohistochemical staining of p53 protein in human lung tissue**. Positive p53 PN II and AM and negative p53 LYM in (A) representative COPD smoker, and (B) non-COPD smoker. **(C)**: Quantitative analysis (mean ± SD) of p53 expression ratio (positive cells/total cells) in three different cell types (PN II, AM, LYM). (** p < 0.05)

Figure 3A shows p53 immunostaining of PN II, AM and LYM in a lung tissue section from a COPD smoker and figure 3B in a non-COPD smoker. The ratio of p53 positive PN II cells (p53 positive PN II/total PN II) was statistically significant higher in COPD patients compared to non-COPD smokers (36% versus 10%, p = 0.01), (figure 3C). On the contrary, the ratio of p53 positive AM cells (p53 positive AM/total AM) and the ratio of p53 positive LYM (p53 positive LYM/total LYM) was not statistically significant different between the two groups (25% versus 10%, p = 0.07 and 6% versus 8%, p = 0.5, respectively), (figure 3C).

#### Bcl2 immunostaining

**Figure 4:**
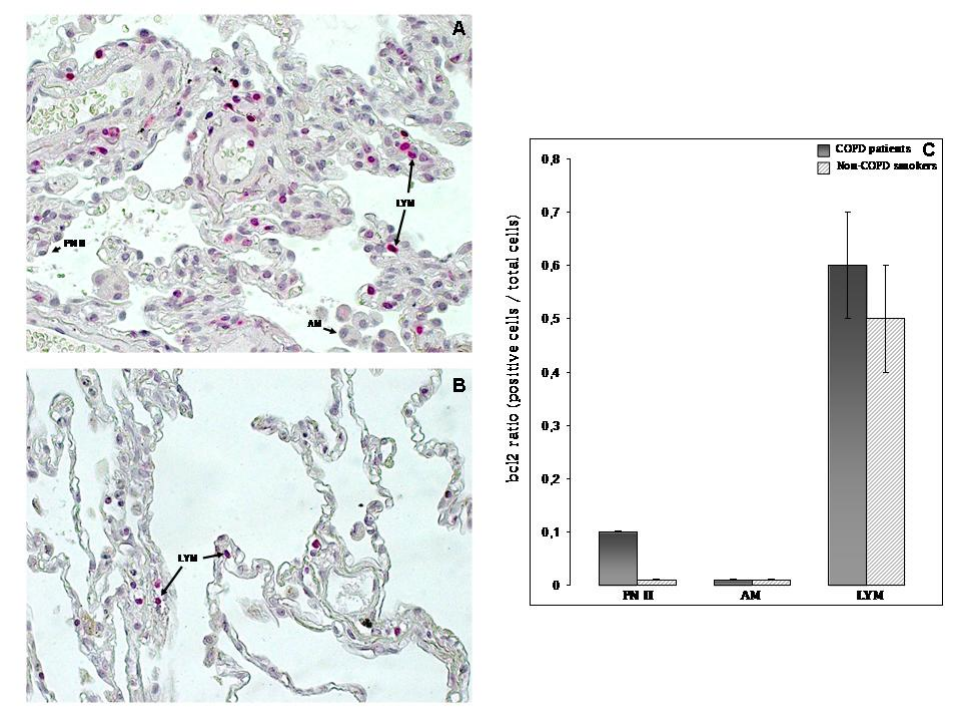
**Immunohistochemical staining of bcl2 protein in human lung tissue**. Positive bcl2 LYM and negative bcl2 PN II and AM (black arrows) in: (A) representative COPD smoker and (B): representative non-COPD smoker. **(C)**: Quantitative analysis (mean ± SD) of bcl2 expression ratio (positive cells/total cells) in three different cell types (PN II, AM, LYM).

Bcl2 was faintly expressed in PN II in COPD patients while no expression was detected in AM in both study groups (Figure 4A, 4B). Bcl2 was expressed in LYM of COPD and non-COPD smokers, but the ratio of bcl2 positive LYM (bcl2 positive LYM/total LYM) did not differ significantly between smokers with or without COPD (0.6 ± 0.1 versus 0.5 ± 0.1, p = 0.5), (Figure 4C).

## Discussion

The present study demonstrated an over-expression of the pro-apoptotic protein p53 in lung tissue of patients with COPD compared with non-COPD smokers, not counterbalanced by the anti-apoptotic protein bcl2. To the best of our knowledge this is the first study to evaluate, at the same time, p53 and bcl2 expression in lung tissue from smokers with or without COPD, by two different techniques. Our results validate and extend observations made by others [3,16,21-26] of an apoptotic imbalance in COPD investigating two apoptosis-related proteins.

Our data as revealed by western blot analysis, showed statistically significant increased p53 protein levels in COPD patients compared to non-COPD smokers (Figure 2), while the immunohistochemistry revealed increased p53 ratio in PN II in COPD patients (Figure 3) compared to non-COPD smokers. Our results are in agreement with those by Hodge et al [3], reporting increased levels of p53 in airway epithelial cells and T lymphocytes gathered from bronchial brushing and bronchoalveolar lavage from ex and current COPD smokers [3]. On the contrary, protein levels, of the anti-apoptotic mediator bcl2 in COPD patients were faintly expressed in PN II while no expression was detected in AM in both study groups (Figure 4A, 4B). Although Bcl2 was expressed in LYM of both study groups did not reach statistical significance between smokers with or without COPD (Figure 4C), reflecting disequilibrium among pro- and anti-apoptotic mediators in favour of apoptosis in COPD patients.

A recent study by Weaver and Liu [24] in rats after exposure to benzene, a ubiquitous environmental pollutant and a cigarette smoking by-product, showed significant up-regulation of pro-apoptotic p53 in lung epithelia of benzene-exposed rats compared to controls, whereas no statistical difference was found in the expression of bcl2 in airway epithelial cells in both study groups [24]. Other groups describe similar findings with an increase in apoptosis of alveolar epithelial cells in patients with emphysema compared to smokers without COPD [25,26] while the anti-apoptotic protein bcl2 was not detected in either normal or emphysematous lung tissue [25].

Furthermore, our data showed increased but not statistically significant p53 levels in AM of COPD patients as compared to non-COPD smokers (Figure 3). Given that macrophages act as scavengers of apoptotic cells, we would expect higher p53 levels in AM of COPD patients, as a result of increased apoptosis of PN II. However, as several groups previously demonstrated [21,22], AM from patients with COPD are less effective in phagocytosing apoptotic epithelial cells compared to controls [21,22]. It has also been shown that neutrophil elastase cleaves the phosphatidylserine receptor on macrophages, resulting in impaired clearance of apoptotic cells [21]. The altered phagocytic capacity of AM in COPD could further result in defective efferocytosis and accumulation of apoptotic cells. Persistence of apoptotic bodies and subsequent release of their toxic contents can result in tissue damage and chronic inflammation leading to COPD progression [23].

On the other hand, the antiapoptotic molecule bcl2 was not expressed in AM of COPD and non-COPD smokers (Figure 4), which could be related with AM homeostasis implicated in lung defence [21].

p53 ratio was decreased in LYM subpopulation of both study groups (Figure 3C), compared to PN II and AM, while bcl2 ratio was increased only in LYM subpopulation in both study groups, although not statistically significant (Figure 4C). The imbalance between pro-apoptotic p53 and anti-apoptotic bcl2 in LYM in favour of bcl2, could possibly explain the persistence of lymphocyte survival into the lung, leading to chronic release of inflammatory mediators.

Yet, there are limitations in this study that have to be taken into account. First, although the study subjects were well characterized, for feasibility reasons the lung tissue specimens were obtained only from subjects undergoing resection for lung cancer. Although it is known that pulmonary malignancy could affect p53 and bcl2 expression, all subjects included in this study had the same comorbidity (*e.g*. lung cancer). Second, the surgical lung biopsy was not performed in patients with more advanced COPD. This could lead to the underestimation of our results, since our data suggest that such a group would exhibit a higher degree of apoptotic deregulation. Third, a confounding factor could be the differences in treatment between subjects, mainly in regard to corticosteroids. Inhaled corticosteroids generally enhance innate immunity while suppress adaptive immunity, thus enhance the survival of neutrophils and AM, but induce the apoptosis of airway dendritic cells [27,28]. It has been demonstrated that corticosteroids induce apoptosis of airway epithelial cells and eosinophils in asthma [27], while no such data are available in COPD [7,28]. Likewise, most of the studies discussed previously do not discriminate between COPD patients that are treated with inhaled corticosteroids and those who are not [7]. In regard to this study, all COPD patients, were stage II GOLD and were treated accordingly, with an inhaled anticholinergic long or short-acting [1]. Only perioperative (2-3 days before surgery and continued until hospital discharge; 10 days in total) and in order to achieve the best possible baseline function and to prevent postoperative disease-exacerbation, COPD patients received twice-daily low dose of inhaled corticosteroids (Table 1), [29,30]. It is still unclear whether inhaled corticosteroids, in such low doses, are able to play a role in the control of apoptosis and remodelling [31]. There is only one reference [32] mentioning the effect of inhaled corticosteroids on airway inflammation in sputum of healthy volunteers, using as a minimum dose 0.5 mg of the drug [32]. Since, our patients received a much lower dose of inhaled corticosteroids (200 mcg total/day), we assume that our results are not subjective to this limitation. However, more studies are needed to clarify that issue.

Moreover, no data are available for the effects of inhaled steroids on the expression of p53 and bcl2 apoptosis mediators [1,13,15,16,33].

Finally, the two groups were not exact matched for age and were all male (Table 1). Although, studies in experimental animals reported increased apoptosis in peripheral blood T-cells with increasing age [33] studies in humans, investigating this possibility reported no significant changes in apoptosis of airway epithelial cells or BAL-derived T-cells, or sputum neutrophils with aging [34,35]. Yet, to the best of our knowledge there are no reports so far, specifically on the effect of age in p53 and bcl2 in COPD patients, or control smokers. Furthermore, studies report no significant differences in the levels of apoptosis or cytokine production between males and females [36].

In conclusion, increased p53 expression in PN II of COPD smokers may contribute to reduced integrity of alveolar septa, resulting in cellular homeostasis defects. In contrast, elevation of anti-apoptotic bcl2 in LYM of COPD smokers could explain the auto-maintenance of the "abnormal" inflammation in COPD. Nonetheless, more studies need to be carried out in order to delineate the above conclusions.

## Abbreviations

COPD: chronic obstructive pulmonary disease; PN II: type II pneumocytes; AM: alveolar macrophages; LYM: lymphocyte-like cells.

## Competing interests

The authors declare that they have no competing interests.

## Authors' contributions

MS has contributed to the acquisition of data and carried out the immunoassays. AVK carried out the immunoassays. EN carried out the molecular genetic studies. EV has contributed to the acquisition of data. MP and NS performed the statistical analysis and contributed to the interpretation of data. NP and SS have contributed to the acquisition of data and subject recruitment. NMS has contributed to interpretation of data and has revised critically the article. EGT has contributed to conception and design of the study, analysis and interpretation of data and has drafted the submitted article. All authors read and approved the final manuscript.

## References

[B1] Rabe KF Hurd S Anzueto A Barnes PJ Buist SA Calverley P Fukuchi Y Jenkins C Rodriguez-Roisin R van Weel C Zielinski J Global Initiative for Chronic Obstructive Lung Disease Global Strategy for the Diagnosis, Management, and Prevention of Chronic Obstructive Pulmonary Disease. GOLD executive summary Am J Respir Crit Care Med 2007 176 6 532 55 10.1164/rccm.200703-456SO 17507545

[B2] Hodge S Hodge G Holmes M Reynolds P Increased peripheral blood T-cell apoptosis and decreased Bcl-2 in chronic obstructive pulmonary disease Immunology and Cell Biology 2005 83 160 10.1111/j.1440-1711.2005.01317.x 15748212

[B3] Hodge S Hodge G Holmes M Reynolds PN Increased airway epithelial and T-cell apoptosis in COPD remains despite smoking cessation Eur Respir J 2005 25 3 447 54 10.1183/09031936.05.00077604 15738287

[B4] Plataki M Tzortzaki E Rytila P Makris D Koutsopoulos A Siafakas NM Apoptotic mechanisms in the pathogenesis of COPD Internat J COPD 2006 1 2 161 171 10.2147/copd.2006.1.2.161 PMC2706617 18046893

[B5] Demedts IK Demoor T Bracke KR Joos GF Brusselle GG Role of apoptosis in the pathogenesis of COPD and pulmonary emphysema Respir Res 2006 7 53 10.1186/1465-9921-7-53 16571143 PMC1501017

[B6] Hodge S Hodge G Holmes M Apoptosis in COPD Curr Respir Med Reviews 2005 1 33 41

[B7] Park JW Ryter SW Choi AM Functional significance of apoptosis in chronic obstructive pulmonary disease COPD 2007 4 4 347 53 10.1080/15412550701603775 18027162

[B8] Schuler M Green DR Mechanisms of p53-dependent apoptosis Biochem Soc Trans 2001 29 684 8 10.1042/BST0290684 11709054

[B9] Haupt S Berger M Goldberg Z Haupt Y Apoptosis - the p53 network J Cell Sci 2003 116 4077 85 10.1242/jcs.00739 12972501

[B10] Martin DA Elkon KB Mechanisms of apoptosis Rheum Dis Clin North Am 2004 30 3 441 54 10.1016/j.rdc.2004.04.008 15261335

[B11] Weaver CV Liu S-P Differentially expressed pro- and anti-apoptogenic genes in response to benzene exposure: Immunohistochemical localization of p53, Bag, Bad, Bax, Bcl-2 and Bcl-w in lung epithelia Exper Toxicol Pathol 2008 59 265 272 10.1016/j.etp.2007.02.012 18093815

[B12] Tzortzaki E Siafakas N A new hypothesis for the initiation of COPD Eur Resp J 2009 34 2 310 5 10.1183/09031936.00067008 19648516

[B13] Agusti A MacNee W Donaldson K Cosio M Hypothesis: does COPD have an autoimmune component? Thorax 2003 58 832 834 10.1136/thorax.58.10.832 14514931 PMC1746486

[B14] Kasahara Y Tuder RM Cool CD Endothelial cell death and decreased expression of vascular endothelial growth factor and vascular endothelial growth factor receptor 2 in emphysema Am J Respir Crit Care Med 2001 163 737 744 11254533 10.1164/ajrccm.163.3.2002117

[B15] Tuder RM Zhen L Cho CY Oxidative stress and apoptosis interact and cause emphysema due to vascular endothelial growth factor receptor blockade Am J Respir Cell Mol Biol 2003 29 88 97 10.1165/rcmb.2002-0228OC 12600822

[B16] Hodge S Hodge G Holmes M Apoptosis in COPD Current Respiratory Medicine Reviews 2005 1 33 41 10.2174/1573398052953668

[B17] Jiménez-Ruiz C Miravitlles M Sobradillo V Gabriel R Viejo JL Masa JF Fernández-Fau L Villasante C Can cumulative tobacco consumption, FTND score, and carbon monoxide concentration in expired air be predictors of chronic obstructive pulmonary disease? Nicotine Tob Res 2004 6 4 649 53 10.1080/14622200410001727948 15370161

[B18] Vlachaki EM Koutsopoulos AV Tzanakis N Neofytou E Siganaki M Drositis I Moniakis A Schiza S Siafakas NM Tzortzaki EG Altered surfactant protein-A (SP-A) expression in type II pneumocytes in COPD Chest 2010 137 1 37 45 10.1378/chest.09-1029 19741063

[B19] Mascaretti RS Mataloun MM Dolhnikoff M Rebello CM Lung morphometry, collagen and elastin content: changes after hyperoxic exposure in preterm rabbits Clinics 2009 64 11 1099 104 10.1590/S1807-59322009001100010 19936184 PMC2780527

[B20] Ikeda K Monden T Kanoh T Tsujie M Izawa H Haba A Ohnishi T Sekimoto M Tomita N Shiozaki H Monden M Extraction and Analysis of Diagnostically Useful Proteins from Formalin-fixed, Paraffin-embedded Tissue Sections J Histochem Cytochem 1998 46 397 403 9487122 10.1177/002215549804600314

[B21] Hodge S Hodge G Scicchitano R Alveolar macrophages from subjects with chronic obstructive pulmonary disease are deficient in their ability to phagocytose apoptotic airway epithelial cells Immunol Cell Biol 2003 81 289 296 10.1046/j.1440-1711.2003.t01-1-01170.x 12848850

[B22] Bratton DL Henson PM Autoimmunity and apoptosis: refusing to go quietly Nat Med 2005 11 26 27 10.1038/nm0105-26 15635442

[B23] Henson PM Cosgrove GP Vandivier RW Apoptosis and Cell Homeostasis in Chronic Obstructive Pulmonary Disease Proc Am Thorac Soc 2006 3 512 518 10.1513/pats.200603-072MS 16921132 PMC2647642

[B24] Weaver CV Liu SP Lu JF Lin BS The effects of benzene exposure on apoptosis in epithelial lung cells: localization by terminal deoxynucleotidyl transferase-mediated dUTP-biotin nick end labeling (TUNEL) and the immunocytochemical localization of apoptosis-related gene products Cell Biol Toxicol 2007 23 3 201 20 10.1007/s10565-006-0165-2 17171516

[B25] Imai K Mercer BA Schulman LL Sonett JR D'Armiento JM Correlation of lung surface area to apoptosis and proliferation in human emphysema Eur Respir J 2005 25 250 258 10.1183/09031936.05.00023704 15684288

[B26] Yokohori N Aoshiba K Nagai A Increased levels of cell death and proliferation in alveolar wall cells in patients with pulmonary emphysema Chest 2004 125 626 632 10.1378/chest.125.2.626 14769747

[B27] de Souza PM Lindsay MA Apoptosis as a therapeutic target for the treatment of lung diseases Curr Opin Pharmacol 2005 5 232 237 10.1016/j.coph.2005.01.012 15907908

[B28] Schleimer RP Glucocorticoids Suppress Inflammation but Spare Innate Immune Responses in Airway Epithelium Proc Am Thorac Soc 2004 1 222 230 10.1513/pats.200402-018MS 16113438

[B29] Jenkins CR Jones PW Calverley PM Celli B Anderson JA Ferguson GT Yates JC Willits LR Vestbo J Efficacy of salmeterol/fluticasone propionate by GOLD stage of chronic obstructive pulmonary disease: analysis from the randomised, placebo-controlled TORCH study Respir Res 2009 10 59 19566934 10.1186/1465-9921-10-59 PMC2714501

[B30] Tashkin DP Celli B Senn S Burkhart D Kesten S Menjoge S Decramer M UPLIFT Study Investigators A 4-year trial of tiotropium in chronic obstructive pulmonary disease N Engl J Med 2008 359 15 1543 54 10.1056/NEJMoa0805800 18836213

[B31] Vignola AM Riccobono L Profita M Foresi A Di Giorgi R Guerrera D Gjomarkaj M Di Blasi P Paggiaro PL Effects of low doses of inhaled fluticasone propionate on inflammation and remodelling in persistent-mild asthma Allergy 2005 60 12 1511 7 10.1111/j.1398-9995.2005.00827.x 16266383

[B32] Alexis NE Lay JC Haczku A Gong H Linn W Hazucha MJ Harris B Tal-Singer R Peden DB Fluticasone propionate protects against ozone-induced airway inflammation and modified immune cell activation markers in healthy volunteers Environ Health Perspect 2008 116 6 799 805 10.1289/ehp.10981 18560537 PMC2430237

[B33] Pahlavani MA Vargas DA Aging but not dietary restriction alters the activation-induced apoptosis in rat T cells FEBS Lett 2001 491 114 118 10.1016/S0014-5793(01)02184-6 11226431

[B34] Hodge S Hodge G Holmes M Reynolds PN Increased airway epithelial and T-cell apoptosis in COPD remains despite smoking cessation Eur Respir J 2005 25 447 454 10.1183/09031936.05.00077604 15738287

[B35] Makris D Vrekoussis T Izoldi M Alexandra K Katerina D Dimitris T Michalis A Tzortzaki E Siafakas NM Tzanakis N Increased apoptosis of neutrophils in induced sputum of COPD patients Respir Med 2009 103 8 1130 5 10.1016/j.rmed.2009.03.002 19329291

[B36] Hodge SJ Hodge GL Reynolds PN Scicchitano R Holmes M Increased production of TGF-beta and apoptosis of T lymphocytes isolated from peripheral blood in COPD Am J Physiol Lung Cell Mol Physiol 2003 285 2 L492 9 12851215 10.1152/ajplung.00428.2002

